# A Review on In Vivo Research Dehydration Models and Application of Rehydration Strategies

**DOI:** 10.3390/nu16203566

**Published:** 2024-10-21

**Authors:** Boyuan Wang, Xiaolu Wei, Xiyan Zhao, Weimin Wang, Jianjun Deng, Haixia Yang

**Affiliations:** 1 College of Food Science and Nutritional Engineering, China Agricultural University, Beijing 100083, China; 2 State Key Laboratory of Vegetable Biobreeding, Institute of Vegetables and Flowers, Chinese Academy of Agricultural Sciences, Beijing 100081, China; dengjianjun@nwu.edu.cn

**Keywords:** dehydration, administration, in vivo models, rehydration strategies

## Abstract

**Background:** Dehydration, a common condition where the amount water lost from the body exceeds intake, disrupts metabolic processes and negatively impacts health and performance. Rehydration, the process of restoring body fluids and electrolytes to normal levels, is crucial for maintaining physiological health. In vivo dehydration models are experimental systems used to study the effects of dehydration on living organisms. However, a comprehensive summary of in vivo models and the application of human rehydration strategies is lacking. **Methods:** This review provides a comprehensive overview of various in vivo models and rehydration strategies. **Results**: In vivo models, stimulated by fluid restriction, exercise, thermal exposure, and chemicals, have been used to study dehydration. Importantly, the principles, characteristics, and limitations of the in vivo models are also discussed, along with rehydration administration methods, including oral, intestinal, intravenous, subcutaneous, and intraperitoneal routes. Additionally, rehydration strategies and the application for managing different dehydration conditions both in daily life and clinical settings have been summarized. **Conclusions:** Overall, this review aims to enhance the understanding of the conditions in which in vivo dehydration models and rehydration strategies are applicable, thereby advancing research into the physiological and pathological mechanisms of dehydration and supporting the development of effective rehydration therapies.

## 1. Introduction

Water is an essential macronutrient, comprising about 60% of the total body weight of healthy individuals with a normal body mass index [1,2]. Dehydration, commonly understood as the excessive loss of body fluids, lacks a universally accepted definition. According to Lacey et al., dehydration can be categorized into different types. Hypertonic dehydration, or water-loss dehydration, is defined as, primarily, an uncompensated deficiency of pure water, leading to increased osmolality in the extracellular compartment, which becomes hypertonic compared to the intracellular space. This condition often results from inadequate water intake or excessive sweating. Isotonic dehydration, also known as salt-loss dehydration, involves the loss of both water and salts proportionately, keeping the osmolality of the extracellular fluid stable and isotonic to the intracellular space. This type is frequently caused by diuretic use or secretory diarrhea. In cases of severe salt loss, hypotonic dehydration may also develop [3].

Recent data revealed concerning hydration levels worldwide. In urban China, only 45% of children, 36% of adolescents, and 28% of adults meet the adequate intake of fluids recommended by the Chinese Nutrition Society [4]. In Europe, 20% of surveyed individuals from Germany, Spain, and Greece were dehydrated based on daily water intake and urine and blood analysis [5]. The situation is particularly severe in Africa, where 93% of Zambian children and 78% of Malian children are dehydrated, as indicated by urine tests [6].

Dehydration was shown to affect 16% of those aged 20–29, 26% of those aged 50–69, and 28% of those aged 70–90 in the United States (US) National Health and Nutrition Examination Survey (NHANES) III cohort. The Established Populations for Epidemiologic Studies of the Elderly (EPESE) study by Duke University found that 21% of individuals aged 70 and above presented with water-loss dehydration [7]. Elderly residents in nursing homes are particularly vulnerable, with 31% of California nursing home residents reported to experience dehydration. Among these, 6% required intravenous (IV) rehydration, and 11% were hospitalized due to dehydration [8].

Dehydration poses several physiological risks, particularly when fluid loss exceeds 3% of the body weight [1]. Early symptoms include thirst, lightheadedness, dry mouth, fatigue, dark and pungent urine, and reduced urination frequency. Dehydration also diminishes muscle strength and endurance, and even a 1–2% fluid loss impairs cognitive function, memory, and psychomotor skills [9]. Children with a 4% fluid deficit may perform poorly on cognitive tasks and exhibit symptoms, such as headaches, irritability, fatigue, and increased respiratory rates [10]. Fluid losses exceeding 8% can be life-threatening [11].

Thus, rehydration, the process of restoring body fluids and electrolytes to normal levels, is essential for maintaining bodily functions. It can be achieved through daily practices as well as clinical interventions. Therefore, the development of dehydration models is crucial for assessing the effectiveness of rehydration treatments under various conditions.

Dehydration models are established as in vivo and in vitro models. In vitro dehydration models are used in controlled laboratory environments and typically involve isolated cells, tissues, or biochemical compounds. Studies have shown that the human keratinocyte cell line (HaCaT) can be dehydrated by drying the culture medium, leading to the production of reactive oxygen species [12]. Another study employed ethanol and acetone gradients, as well as low-temperature dehydration methods, to dehydrate mice aortas and renal glycocalyxes [13]. These models allow for precise experimental control and offer a valuable insight into cellular responses, molecular pathways, and dehydration-related mechanisms. However, they lack the complexity of whole-organism interactions and systemic responses.

In contrast, in vivo dehydration models allow for the examination of dehydration in living organisms, such as animal models or human volunteers. These studies allow researchers to assess the effects of dehydration on various organ systems, observe systemic responses, and evaluate rehydration methods under more natural conditions. This approach replicates dehydration within specific contexts, aiding the exploration of underlying mechanisms and the development of targeted treatments for dehydration-related disorders. However, comprehensive reviews on in vivo dehydration models for research are lacking, and no systematic summary currently exists on the application of human rehydration strategies.

This review aims to provide a detailed analysis of in vivo dehydration models, including their construction, benefits, drawbacks, and applications. It serves as both an experimental framework for laboratories and a foundation for developing tailored rehydration therapies suited to specific dehydration scenarios. Additionally, we discuss daily rehydration strategies and clinical therapies, offering insight for developing commercial hydration products.

**Figure 1:**
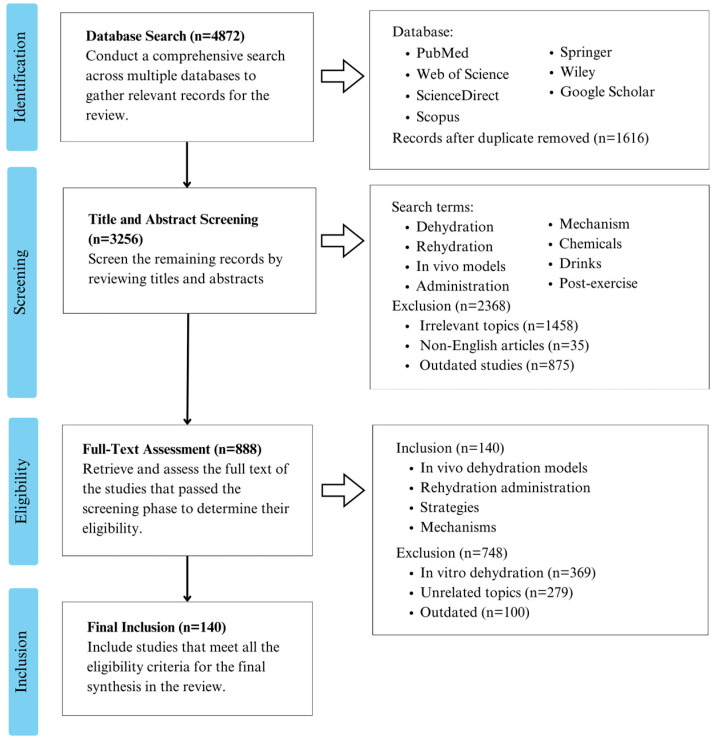
Flowchart of the literature screening steps used for the review.

The scientific literature for this review on in vivo dehydration models and rehydration strategies was sourced from a range of databases, including PubMed, Web of Science, ScienceDirect, Scopus, Springer, Wiley, and Google Scholar. The search terms encompassed “dehydration”, “rehydration”, “in vivo models”, “administration”, “mechanism”, “chemicals”, “beverages”, and “post-exercise”. Only articles written in English were considered, with selections based on an initial review of the titles and abstracts. Potentially relevant articles were further reviewed in full to determine their eligibility. The inclusion criteria were studies that addressed in vivo dehydration models, rehydration methods, strategies, or mechanisms. The exclusion criteria included research focusing on in vitro models, irrelevant subjects, or outdated studies (Figure 1).

## 2. Dehydration Models

Dehydration can be induced in research models using various methods, including fluid restriction, exercise, thermal exposure, and chemical induction. Each of these methods is designed to replicate specific dehydration scenarios, which is crucial for understanding how organisms respond to water loss and developing strategies to address dehydration-related health issues.

### 2.1. Fluid-Restriction-Induced Dehydration Models

Fluid restriction by withholding water from subjects is the simplest method for inducing dehydration. This method can be applied to both animal and human models, with different approaches used to induce either acute or chronic dehydration. The fluid restriction model mimics scenarios where fluid intake is insufficient to meet physiological demands, such as prolonged periods without access to water or inadequate consumption due to a reduced sense of thirst or mobility limitations, commonly seen in elderly people and infants.

Acute dehydration involves the rapid induction of systemic dehydration in animals through continuous water deprivation over a specific period. The duration and protocols vary depending on the animal model used.

Currently, rats are the most common laboratory animals used to study fluid homeostasis and motivational stimulation. Typically, water-restricted rats experience a 6% body weight loss (BWL) for mild dehydration, 6–10% BWL for moderate dehydration, and >10% BWL for severe dehydration. In Sprague Dawley rats, mild dehydration occurs between 18 and 24 h of water deprivation, moderate dehydration between 36 and 48 h, and severe dehydration between 66 and 72 h [14]. Humane considerations, as recommended by animal welfare associations, advise against extending dehydration beyond 72 h [15].

In mice, noticeable physical or behavioral signs of dehydration emerge after 24 h of water deprivation, with BWL reaching 12%. After 48 h of water deprivation, the BWL increases to 18%, surpassing the humane endpoint threshold of 15% [16]. Acutely deprived mice lose a larger proportion of body weight per unit time than larger mammals [16]. In addition to BWL, a five-point scale (Table 1) is used to assess the dehydration status of mice, with attitude and appearance scores declining significantly as dehydration duration increases. Water-deprived mice also exhibit increases in plasma osmolality, plasma sodium, plasma corticosterone, total plasma protein, hematocrit (Hct), and blood urea nitrogen (BUN) levels, as well as platelet counts, packed-cell volume (PCV), urinary sodium and potassium levels, urinary osmolality, and plasma renin activity (PRA), among others. These mice also exhibit reduced food intake, white blood cell counts, and plasma volume. Changes in plasma osmolality and volume reflect the increase in extracellular fluid osmolality as circulating fluid decreases during dehydration. Reduced water intake leads to fluid loss from both the intracellular and extracellular compartments, increasing Hct and total plasma protein and signaling alterations in the extracellular fluid. Simultaneously, changes in the intracellular environment affect the activity of other hormones and enzymes, impacting overall physiological function. PRA indicates the body’s maintenance of fluid volume through the renin–angiotensin system. As body fluid volume declines, vasoconstriction helps maintain blood pressure and stimulates aldosterone release. Aldosterone reduces sodium excretion by the proximal tubules, preserving plasma sodium and promoting fluid retention through cellular dehydration. A significant increase in PRA signals the body’s growing difficulty in maintaining fluid volume. Reduced food intake, known as dehydration-induced anorexia, helps lower the body’s solute load and acts as a compensatory response to dehydration [16].

In our review, we identified body weight, serum sodium, serum chloride, serum potassium, plasma volume, plasma osmolality, and urine osmolality as the most frequently utilized parameters in studies assessing hydration status. Therefore, these will be referred to as “common indicators” throughout the subsequent sections.

Several studies have employed dogs to establish dehydration models. Beagle dogs aged 9–14 months were subjected to 17 h of food and water deprivation, which resulted in increased neutrophil counts and decreased lymphocyte counts [17]. In another experiment, female beagles aged 1–8 years were subjected to 12 h of water deprivation [18]. Healthy greyhounds aged 5 years were subjected to restriction from water for up to 24 h to induce hyperosmotic dehydration, which led to significant common indicator change [19]. Dehydration was assessed through the dogs’ physical signs (Table 2), including general inspections, evaluations of respiration and pulse rate, rectal body temperature recordings, mucous membrane examinations, and the skin turgor test (STT) [20].

Chronic dehydration models are established by continuously restricting water intake, which prevents subjects from achieving adequate hydration levels. Two main methods are used to establish a chronic dehydration model. One involves consistently providing less water than the experimental animals require. For example, after measuring the daily water intake of rats, they were provided with only 75% or 50% of their normal intake for 7 days. Dehydration levels were then assessed through weight measurements and behavioral evaluations [21]. CD1 mice subjected to 7 days of 50% and 75% water restriction exhibited declines in both appearance and attitude scores. The BWL in the 75% and 50% groups was approximately 5% and 11%, respectively. Water restriction also led to increases in plasma osmolality, Hct, renin activity, corticosterone levels, and total protein levels compared to the control group [16].

Intermittent water restriction, another method for inducing chronic dehydration, is characterized by periodic water deprivation that causes fluctuations in hydration status. In one protocol, rats were subjected to water deprivation for 15 h each evening over a 20-day period [22]. Another experiment involved long-term repeated dehydration, where rats underwent 10 h of water restriction every 24 h, 5 days a week, over 4 weeks. This resulted in a final 15% BWL and increased plasma osmolality [23].

Human volunteers are suitable for building models of acute dehydration. In such studies, the participants were required to abstain from fluid intake for 13, 24, and 37 h, and dehydration was induced by only allowing the consumption of foods with low water content, resulting in 1.0%, 1.8%, and 2.7% BWL, respectively. The participants experienced a reduction in urine volume, accompanied by an increase in angiotensin II levels and changes in the common indicators. Typically, urine sodium, potassium, and chloride levels decrease under these conditions; although, they peak at 24 h of dehydration. The study also utilized subjective sensations, such as thirst and headache, as indicators of dehydration [24]. The onset of thirst is associated with angiotensin II. In response to dehydration, the activation of the sympathetic nervous system causes vasoconstriction and enhances renal water reabsorption. The release of renin stimulates the production of angiotensin II, which, in turn, increases thirst [25].

Fluid restriction is generally the simplest and most cost-effective way to induce dehydration. However, precisely controlling individual water intake in chronic dehydration experiments with animals can be challenging. The literature review yielded relatively few studies on fluid restriction-induced dehydration models in human volunteers, likely due to ethical considerations. While human volunteers offer an advantage over animal models in studying human-relevant physiological changes due to dehydration, long-term water deprivation in human subjects poses potential health risks and should be approached with caution.

### 2.2. Exercise-Induced Dehydration Models

Exercise-induced dehydration models simulate the conditions faced by individuals involved in prolonged or high-intensity physical activities, where fluid loss through sweating exceeds intake and is compounded by increased metabolic heat. These models reflect real-life situations encountered by athletes and outdoor workers. Both animal models and human participants can be used for these studies.

Rats can become dehydrated through treadmill exercise. Male albino rats weighing between 130 and 150 g were acclimated to treadmill activity over 3 days before experimentation. Then, they performed four sets of treadmill exercises at a speed of 25 m/min, with 2 min intervals between each set. Following dehydration, these rats exhibited elevated levels of potassium, calcium, phosphorus, creatine phosphokinase (CPK), lactate dehydrogenase (LDH), serum urea, and creatinine and decreased sodium, chloride, magnesium, and aldosterone levels. These changes reflect electrolyte imbalances and oxidative stress [26].

This model allows for precise control over exercise duration and intensity. However, it does not eliminate the possibility of interference among test subjects, and reduced activity levels may occur. Notably, research on exercise-induced dehydration in animal models is limited, likely because human volunteers can also undergo this type of dehydration, offering more directly applicable insight but potential harm, raising ethical concerns. Since exercise is a common cause of dehydration, future studies should focus on developing standardized animal models.

In human studies, exercise-induced dehydration is achieved through three methods, one of which involves fixed-intensity and fixed-duration exercise protocols. In two previous studies, the participants cycled for 1–1.5 h at 50–60% of their maximum heart rate in an environment heated to 30–32 °C, without consuming fluids. The indicators of dehydration included a 1.2% BWL, a urine-specific gravity of ≥1.020, and a urine color scale reading above 4. Pérez-Luco at el. used a decrease in the saliva flow rate to assess dehydration [27,28,29]. Another study involved participants completing three sets of 25 min intermittent-intensity exercises on a treadmill, stationary bike, and elliptical machine in any sequence, with 5 min rest periods between the sets and no fluid intake. The goal was to achieve a 2.5–3% reduction in body weight, using changes in body weight, urine volume, and sweat sodium concentration as hydration indicators [30].

Exercise-induced dehydration models can also be constructed by having participants exercise until a predetermined percentage of BWL is achieved. In one study, the participants exercised on a friction-braked cycle ergometer at an intensity of 2 W/kg body mass in a 35 °C environment, with each session lasting 10 min, followed by a 5 min rest. This cycle was repeated until a 1.6% BWL was achieved, with urine volume and osmolality used as dehydration markers [31]. In another study, participants cycled on a Monark cycle ergometer at 60 rpm in a 38 °C climate chamber until they reached a 3–5% BWL, resulting in elevated serum osmolality [32].

Some models combine fluid restriction with exercise-induced dehydration. In a study by Hackney et al., the participants walked on a motorized treadmill at 5.6–6.4 km/h on a 2% incline for 25 min, followed by a 5 min rest, and repeated this cycle six times. Fluid intake was restricted for 14 h before the experiment. This approach validated the thirst perception and the thirst sensation scales as effective measures of hydration status postexercise [33].

Besides serum and urine osmolality, tear fluid osmolality has also been shown to be a noninvasive measure of hydration status postexercise. However, due to significant interindividual variability, its effectiveness was only validated at the group mean level [34].

Compared to animal models, in vivo dehydration studies involving human volunteers provide more relevant insights for human dehydration research. Human participants also tend to be more cooperative during these studies. However, these studies raise ethical concerns, particularly regarding the potential harm or discomfort from dehydration. In a study by Hackney et al., the participants experienced dehydration, resulting in a 5% BWL, raising concerns about the participants’ health and safety. Another common drawback of exercise-induced dehydration is the need for exercise equipment, which is often expensive.

### 2.3. Thermal Dehydration Models

Thermal dehydration is induced by exposing experimental animals to elevated temperatures, leading to dehydration, as they use evaporative heat loss mechanisms, such as panting, sweating, and saliva spreading, to maintain core body temperature. These models simulate dehydration conditions caused by prolonged exposure to extreme heat. Rats and mice are commonly used as thermal dehydration animal models. In studies with rats, the animals are typically placed in an environment at 40 °C for 0–4 h without access to water. Rats subjected to heat-induced dehydration showed increased water intake, plasma osmolality, plasma sodium, hematocrit, and plasma protein levels, along with decreased plasma potassium levels and urine output [35,36]. Another method involves using an infrared lamp to gradually raise the colonic temperature by 0.05 °C/min for 60 min or until it reaches 41.5 °C. Colonic temperature is measured with a thermistor probe inserted into the rat’s anal sphincter. This procedure results in weight loss and increased plasma osmolality and sodium levels [37].

In mice, dehydration is induced by placing them in a 37 °C environment with 15–20% relative humidity, without access to water, which leads to a 4–11% BWL within 5–10 h [38].

While this method is relatively straightforward, caution is necessary to avoid exposing animals to excessively high temperatures that may cause undue harm. Additionally, research on heat-induced dehydration is limited, with some studies, such as those by Barney et al. [35,36], being outdated and of lower current relevance. Therefore, optimizing this dehydration model based on the recent literature may be necessary.

### 2.4. Chemical-Induced Dehydration Models

Chemical-induced dehydration models are developed by exposing subjects to specific chemical agents, such as saline, isoproterenol, polyethylene glycol (PEG), angiotensin II (ANG II), furosemide, and captopril.

#### 2.4.1. Saline Treatment

The administration of hypertonic saline, either by injection or ingestion, can lead to hypertonic dehydration. This condition disrupts osmolality regulation and raises serum sodium levels, leading to intracellular dehydration, as water is drawn out of cells due to the osmotic gradient. Hypertonic saline is typically administered through one of four methods: subcutaneous injection (SC), intracerebroventricular injection (ICV), intraperitoneal injection (IP), or orally.

SC injections of saline in mice can induce cellular dehydration, leading to a significant increase in water intake. For instance, administering 0.5 mL of 3% or 6% weight/volume hypertonic saline to 60-day-old male C57BL/6J mice was shown to cause a dose-dependent increase in water consumption [39].

ICV injections of hypertonic saline have been shown to be dipsogenic in mice. This method involves administering hypertonic saline directly into the lateral ventricle under sterile conditions, typically as a 2 μL bolus of 600 mM/μL hypertonic saline [39]. While this technique offers precise control over the experimental variables, it is complex and carries significant risks, including potential injury or death to the animals.

IP injections of hypertonic saline can also induce dehydration. Adult albino mice weighing 20–30 g demonstrated a marked increase in water intake after receiving 10 mL IP injection of 1 M NaCl per kg body weight [40].

The oral administration of hypertonic saline can induce cellular dehydration accompanied by hypernatremia and hyperosmolality, with minimal impact on extracellular volume. This suggests that hypertonic saline prompts fluid to move into the gut lumen, offsetting fluid movement from the cells, thereby mitigating changes in the extracellular space. Oral saline-induced dehydration methods can be categorized into short-term and long-term models. In the short-term model, rats were deprived of food and water then administered 2 mL of 2 mol/L NaCl via gavage after acclimatization to water gavage. After 1 h of intragastric administration, the rats developed a 6% increase in plasma sodium levels and a 5% increase in plasma osmolality [41,42].

The long-term oral administration of saline also induces moderate cellular dehydration. In one study, rats received 3 mL of a 1.5% NaCl solution by gavage three times daily for 20 days [43]. Another study examined dehydration-induced anorexia in rats that had free access to food and a 2.5% NaCl solution for drinking over 7 days, with body weight, liquid, and food intake monitored daily as indicators [44]. In another study, male Sprague Dawley rats aged 5–6 weeks developed chronic salt load dehydration after consuming water containing 2% NaCl for 6–8 days [45].

#### 2.4.2. Isoproterenol Treatment

SC isoproterenol administration stimulates water consumption by enhancing the activity of the peripheral renin–angiotensin system. The dosage for C57BL/6 mice ranges from 15 to 30 μg/kg. Water intake was monitored for 90 min after isoproterenol injection and showed dose-dependency [39]. However, compared to hypertonic saline, even high doses of isoproterenol (50–400 μg/kg) in CD1 mice resulted in only modest drinking, indicating that isoproterenol is not an ideal dipsogen and induces only mild dehydration [40]. Thus, it is unsuitable for studies requiring severe dehydration.

#### 2.4.3. Polyethylene Glycol (PEG) Treatment

PEG induces water consumption by causing hypovolemia, which, in turn, stimulates renin release [40]. Studies administered PEG (25% weight/volume, 1 mL/mouse) subcutaneously to mice, with water intake typically monitored for 2–6 h [39,40]. A significant increase in water intake was observed within 2 h after injection [39]. However, the majority of mice became critically ill 24 h after receiving PEG; although, the cause of the high mortality rate was unclear [39]. Therefore, caution is advised when using this treatment method.

#### 2.4.4. Angiotensin II (ANG II) Treatment

Neither acute systemic injections nor chronic systemic infusions of ANG II have consistently increased water consumption [39,40,46,47]. The effects of systemic ANG II on drinking behavior are complex due to its impact on the peripheral vasculature [48,49]. SC injections of ANG II did not enhance water intake, but ICV injections of ANG II were proven effective when administered centrally over an extended period [46]. A 2 μL intracerebroventricular injection of ANG II in doses ranging from 3.1 to 100 ng significantly increased water intake in C57BL/6 mice, with 50 ng inducing the most substantial drinking response [39]. However, additive effects on water intake were not seen in rats and mice treated with combined ICV injections of hypertonic saline and ANG II [39,50].

#### 2.4.5. Furosemide Treatment

Furosemide, a fast-acting diuretic, is commonly used in animal studies to induce dehydration through IP and IV injections. It inhibits the reabsorption of sodium and chloride in the proximal and distal tubules, as well as in the thick ascending loop of Henle. By disrupting the sodium–chloride cotransport system, furosemide promotes the excessive excretion of water, sodium, chloride, magnesium, and calcium [51].

Rabbits receiving an IP injection of 50 mg/mL (5 mg/kg) furosemide experienced systemic dehydration, characterized by a 5% body weight fluid loss, with food and water withheld to ensure accurate modeling. Following dehydration, these rabbits showed increased levels of hemoglobin, packed cell volume, total plasma protein, creatinine, and BUN levels, while sodium and chloride levels decreased [52].

Adult rabbits received furosemide at a dosage mentioned previously via intravenous injection, which produced iso-osmotic dehydration or hypovolemia [53]. Additionally, in rats, a slow IV injection of 8 mg/kg furosemide following the administration of 4.2% hypertonic saline (3.2 mL/100 g) enhanced diuresis and led to changes in the common indicators. It also reduced water content in the brain, lungs, muscles, and small bowel [54].

#### 2.4.6. Captopril Treatment

Captopril is an angiotensin-converting enzyme inhibitor that increases central ANG II levels, leading to fluid imbalance. The chronic administration of 0.5 and 1.0 mg/mL captopril in drinking water to normal C57BL/6 mice significantly increased water intake, while the lowest dosage of 0.1 mg/mL did not significantly elevate water consumption compared to controls. Combined treatment with furosemide and captopril injections did not increase water intake [39].

Chemical-induced dehydration models are generally less time-consuming and involve fewer confounding variables. However, they often require sophisticated techniques, such as intraventricular injections, making the procedures more complex. Some invasive methods may also cause harm to the animals.

In summary, fluid restriction is the simplest and most cost-effective method for inducing dehydration, though it can be time-consuming and challenging to precisely control hydration levels. It effectively simulates dehydration from prolonged low fluid intake in humans. Exercise-induced dehydration, while requiring more complex protocols and specialized equipment, allows for precise control of hydration status through exercise duration and intensity. It is highly translatable to humans, as postexercise dehydration is a common issue. Thermal dehydration is easier to implement than exercise or chemical methods, as it mainly requires temperature control. However, high temperatures can pose risks to subjects. This model mimics dehydration from prolonged exposure to hot environments. Chemical induction, while efficient for short-term studies, involves invasive procedures and extensive training, which may stress subjects. It has limited translatability to humans, as dehydration from excessive medication intake is uncommon. In animal studies, the fluid restriction model is typically sufficient to achieve research objectives; whereas, exercise-induced dehydration models are more commonly employed in human studies. The method choice depends on the specific research requirements and conditions, with each offering distinct advantages and challenges (Table 3).

## 3. Administration for Rehydration in In Vivo Dehydration Models

After inducing dehydration in in vivo models, various research purposes necessitate rehydration, such as studying the physiological effects of dehydration and rehydration or evaluating specific rehydration methods or products. This section reviews the different rehydration administration techniques used in in vivo dehydration models, including oral administration, intestinal perfusion, IV injection, SC injection, and IP injection. The characteristics, benefits, and limitations of each administration are summarized to assist researchers in selecting the most appropriate approach based on their specific research objectives.

### 3.1. Oral Administration

Oral administration is a widely used and effective method for rehydration in dehydration models. In animal models, fluids can be administered via drinking bottles, droppers, or gavage (feeding through an esophageal tube). In human dehydration models, oral rehydration is typically achieved using oral rehydration solutions. Evidence supports the use of plain water, as well as NaCl, glucose, and oral rehydration solutions (ORSs) for fluid replacement [55,56,57,58,59].

When rehydrating experimental animals, allowing them unrestricted access to water, known as ad libitum fluid access, ensures that they can drink according to their physiological needs. This method can be applied to laboratory animals, such as rats and rabbits [60,61]. Typically, the animal drinks until they have consumed enough water. The rehydration time may vary from a few hours to several days, depending on the severity of the dehydration. For instance, in rats severely dehydrated for 5 days, plasma osmolality normalized within 3 days of ad libitum water access, while vasopressin and oxytocin levels stabilized after 14 days [62].

In studies involving human participants, those with exercise-induced dehydration were rehydrated with fluids equivalent to 150% of their BWL within 100 min postexercise or with four equal portions of fluid at 15 min intervals during a 1 h rehydration period [27,31]. Another study required the participants to rest for 45 min after exercise before rehydrating with fluids equivalent to 100% of their BWL, consuming 25% of the total volume every 10 min for the first 20 min and 12.5% every 10 min over the next 40 min [30].

Oral rehydration is generally convenient and labor-saving [63,64]. It is noninvasive and reduces stress and discomfort for animals, while simplifying the process for researchers. Oral administration is also cost-effective, making it suitable for experiments with limited budgets [64]. Palatability issues can be managed through gavage [65], and this method is applicable for rehydration in human dehydration models as well. Overall, oral rehydration effectively meets the needs of most studies.

However, in cases of gastrointestinal compromise or critical illness, oral absorption may be inadequate for rehydration. The effects of oral rehydration are seen more slowly compared to IV methods, making it less suitable for urgent fluid resuscitation in critically ill subjects [66].

### 3.2. Intestinal Perfusion

Intestinal perfusion is a technique used to administer fluids directly into the intestines of dehydrated laboratory animals. It aims to restore hydration and maintain fluid balance. This method involves delivering a rehydration solution through a catheter inserted into the animal’s small intestine, with careful consideration of the infusion rate. Monitoring vital signs, such as heart rate, blood pressure, and urine output, is essential for guiding adjustments to the infusion rate.

Research has demonstrated that small intestinal infusion of the standard World Health Organization Oral Rehydration Solution (WHO-ORS) can rapidly rehydrate experimental rabbits. In one study, after anesthesia, a cannulated jejunal loop was perfused with the rehydration solution at a constant rate of 0.5 mL/min at 37 °C [67].

Intestinal perfusion allows for precise and controlled fluid delivery, enhancing absorption and distribution by bypassing the stomach, which is particularly beneficial when stomach health or function is compromised [63]. This technique can also be used to study gastrointestinal physiology in animal experiments by perfusing specific segments of the intestine with rehydration solutions [67]. It provides valuable insight into how different fluid compositions, infusion rates, and other factors impact intestinal function.

Despite its benefits, intestinal perfusion has certain limitations. The procedure requires the surgical insertion of a catheter into the small intestine, increasing complexity and posing risks, such as infection or tissue damage. Additionally, the technique may interfere with normal gastrointestinal physiology, potentially altering motility, digestion, or other processes. Translating intestinal perfusion from the laboratory to clinical settings is challenged by environmental control and ethical concerns. Furthermore, it is not widely used for rehydrating animal dehydration models, and the supporting evidence is relatively limited, necessitating careful consideration of its use.

### 3.3. Intravenous (IV) Injection

IV injection is widely regarded as the most efficient method for fluid administration in animals [64]. This technique involves delivering fluids directly into the bloodstream through a needle or catheter inserted into a vein. In small laboratory animals, such as rodents, the common sites for IV injections include the tail vein, lateral saphenous vein, or marginal ear vein. In rabbits, the ear or cephalic veins are preferred, while larger animals, such as dogs, cats, pigs, and primates, typically receive IV injections into the cephalic, femoral, or saphenous veins. The administered volume should generally not exceed 5% of the circulating blood volume [65].

In previous studies, dehydration induced in mini pigs using NaCl and NH4Cl was effectively treated with IV rehydration. A fluid mixture containing 2.5% glucose, 42 mM/L NaCl, and 42 mM/L NaHCO3 was administered at 60 mL/kg over 3 h. Subsequently, a remaining solution containing 3.35% glucose, 28 mM/L NaHCO3, and 28 mM/L KCl was infused at an average rate of 220 mL/kg over 21 h [68].

IV injection offers rapid and controlled fluid delivery, which is particularly advantageous in cases of severe dehydration or when immediate fluid balance restoration is required [63]. This method ensures the complete absorption of fluids without the need for gastrointestinal transit, bypassing potential issues related to poor oral intake or absorption [63].

However, IV injection is an invasive procedure, requiring the insertion of a needle or catheter into a vein, which can cause discomfort and distress to the animal, potentially impacting experimental outcomes [63]. In small animals, the procedure is particularly challenging due to the difficulty in locating veins, increasing the risk of failed venous access and prolonging the process.

### 3.4. Subcutaneous (SC) Injection

SC injection involves administering fluids into the SC tissue beneath the skin. Ensuring that the skin at the injection site is loose is important to minimize discomfort. The needle is then gently inserted at an appropriate angle, allowing the fluid to be infused slowly and gradually into the SC space without causing tissue damage [65]. When injecting mammals, trimming the hair at the injection site can reduce interference. For common laboratory animals, the maximum injection dose should not exceed 5 mL/kg per site [65].

According to Animal Care Services at the University of British Columbia, SC, injection is effective for rehydrating small animals, such as rodents. When a large rehydration volume is needed, alternating the injection site on the rodent’s back can help prevent skin discomfort and tension. The University of Montana’s laboratory animal resource guidelines also recommend heating the rehydration solution to approximately 37.7 °C before SC injection in rodents.

SC injection results in a slower absorption rate compared to other parenteral routes, leading to a more prolonged effect. While this method is easier to master than IV delivery, its slower rehydration rate and limited volume capacity may impede rapid fluid resuscitation in cases of severe dehydration. However, it is important to note that the evidence supporting SC injection for rehydrating dehydrated experimental animals is primarily limited to university animal care guidelines. This method has not been extensively validated in reliable studies or literature, so researchers should use it with caution.

### 3.5. Intraperitoneal (IP) Injection

IP injection involves introducing fluids into the peritoneal cavity, the space surrounding the abdominal organs. This technique requires inserting a small needle through the abdominal wall and slowly infusing the liquid solution. The peritoneal membrane efficiently absorbs the fluid, allowing it to enter the bloodstream and distribute throughout the body. However, this method is unsuitable for animals larger than rodents or for pregnant animals, as the needle may puncture the uterus [65]. IP injections should be limited to once per day per animal, with a maximum volume of 10 mL/kg for laboratory animals [65]. The University of Montana’s laboratory animal resource guidelines suggest that SC injections are ineffective when animals are 10% dehydrated, and IP injections should be used for rapid rehydration.

In dehydrated rats that received an IP injection of fluid equivalent to 3% of their body weight, those injected with water and isotonic glucose solution showed a significant reduction in water intake [69]. In another study involving middle cerebral artery occlusion, dehydrated rats were treated with three 1.5 mL IP injections of saline [70].

**Figure 2:**
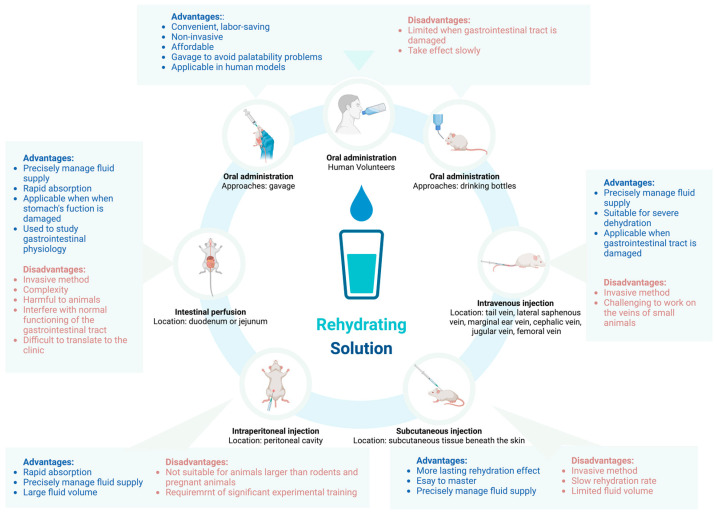
Different administration for rehydration in in vivo dehydration models.

The peritoneal cavity offers a large surface area for fluid absorption, enabling efficient uptake of fluids and electrolytes. IP injection also allows for the administration of larger fluid volumes compared to other routes, making it suitable for situations requiring rapid rehydration. However, the technique can be challenging due to the difficulty in ensuring that fluids are injected into the abdominal cavity rather than the intestines. Proper training is essential to avoid damaging abdominal organs [65] (Figure 2).

## 4. Application of Rehydration Strategies and Involved Mechanisms

The exploration of in vivo dehydration models ultimately aims to guide the development of effective rehydration strategies for humans. In this section, we classify these strategies into two categories: daily rehydration strategies and clinical rehydration strategies, addressing the conditions and severity of dehydration applicable to each. By exploring the applications and mechanisms underlying these strategies, we can better understand how to implement effective rehydration practices to promote overall health and wellbeing in diverse scenarios.

### 4.1. Daily Rehydration Strategies

Dehydration in daily life is commonly caused by factors, such as prolonged heat exposure, high-intensity activities, mild diarrhea, and inadequate daily water intake. Mild dehydration can generally be managed by consuming plain water, beverages, and food. In this section, we will review rehydration strategies suitable for these everyday situations, referring to them as daily rehydration strategies.

#### 4.1.1. Drinking Plain Water

Drinking plain water is the most straightforward and cost-effective rehydration strategy; it is essential for daily life. Water helps to replace fluids lost through normal activities, sweating, and metabolic processes. However, during periods of intense physical activity or significant electrolyte loss, consuming plain water alone may be insufficient for adequate rehydration. Consuming only water under these conditions can lead to decreased plasma osmotic pressure and reduced sodium levels, which, in turn, may increase urine production and diminish the desire to drink, ultimately slowing the rehydration process [71].

#### 4.1.2. Drinking Beverages

Beverages, distinct from plain water, include any drinkable liquids containing substances, such as electrolytes, carbohydrates, proteins, and other nutrients. This category encompasses sports drinks, fruit juices, milk, soda, coffee, tea, and more. Research based on data from the NHANES III database showed that beverages account for approximately 40–45% of total water intake among American adults, highlighting their importance in fluid replenishment [72]. Due to their additional components, beverages often provide superior hydration compared to plain water. Beverages rich in electrolytes, particularly potassium and sodium, and macronutrients are more effective in maintaining fluid balance and supporting long-term hydration. However, beverages containing diuretics, such as caffeine and alcohol, may hinder rehydration [73]. Additionally, energy-dense beverages promote fluid retention more effectively than calorie-free options, as they empty more slowly from the stomach, reducing diuresis [73].

##### Sports Drinks

Sports drinks belong to a broad category, with various brands offering different product formulas. Generally, sports drinks can be categorized as either carbohydrate–electrolyte beverages or noncarbohydrate–electrolyte beverages.

Carbohydrate–electrolyte sports drinks replenish glycogen stores in muscles and the liver, counteract sweat losses, and prevent dehydration [74]. Some formulations also include amino acids to reduce fatigue and enhance muscle function, along with B vitamins to support metabolism and energy production, aiding in postexercise recovery [75]. Ingredients, such as ω-3 polyunsaturated fatty acids, vitamin E, and polyphenols, may be added to promote recovery, minimize oxidative cell damage, and counteract the generation of proinflammatory molecules [75].

Research suggests that the regular consumption of hypotonic (<275 mOsmol kg^−1^) carbohydrate–electrolyte drinks during exercise is more beneficial for fluid recovery than isotonic and hypertonic options [76]. However, despite the marketing claims of various products, limited evidence exists to support the superiority of any one sports drink [74].

##### Milk

Milk, including skim milk, is an excellent rehydration option after exercise and offers a more affordable alternative to sports drinks. A study comparing a range of beverages, including cola, fruit juice, milk, sports drinks, tea, and coffee, showed that milk and skim milk had some of the strongest hydrating properties, with skim milk outperforming whole milk in fluid retention [73].

Milk is rich in potassium, sodium, and protein, making it especially effective for post-exercise muscle protein synthesis. Skim milk, often regarded as the best natural protein drink due to its essential amino acids, can restore fluid balance more effectively after exercise-induced dehydration than carbohydrate–electrolyte sports drinks. Furthermore, milk protein has been shown to be more effective in promoting hydration postexercise compared to an equivalent amount of carbohydrates [73,77].

Chocolate milk, with its added carbohydrates, is also effective in supporting postexercise recovery. Studies suggest that consuming chocolate milk immediately after exercise and again 2 h later is one of the best recovery options, as it helps reduce exercise-induced muscle damage [78].

##### Fruit Juice

Fruit juice is another high-quality beverage that maintains fluid balance. Juices typically contain high water content along with carbohydrates, essential vitamins, and minerals. For example, orange juice is rich in potassium and has been shown to maintain fluid retention significantly better than plain water [73].

Additionally, research suggests that offering half-strength apple juice followed by fluids that children enjoy can effectively treat mild dehydration caused by gastroenteritis [79]. This makes juice a valuable option for hydration as well as for replenishing essential nutrients.

#### 4.1.3. Eating Food

Evidence suggests that rehydration can be supported by water-rich foods and that eating promotes water absorption. Both factors demonstrate that consuming food can enhance the rehydration process. Food plays a significant role in daily hydration, with approximately 25% of the daily fluid intake of American adults coming from the water content in food, according to the Continuing Survey of Food Intakes by Individuals [80]. Consuming foods, particularly those with high water content, such as fresh fruits and vegetables, is an important strategy for maintaining fluid balance. A study of German children found that the regular consumption of fruits and vegetables improved hydration status [81].

Beyond the direct fluid contribution of water-rich foods, eating food during rehydration can enhance the body’s ability to replenish water stores. In one study, participants who were dehydrated from exercise were given either a meal with water or a carbohydrate–electrolyte beverage. The group that consumed the meal and water showed better cumulative urine volume and net fluid balance [82]. Another study demonstrated that dehydrated participants who consumed chicken broth or chicken noodle soup with water experienced better plasma volume recovery compared to those who drank plain water or carbohydrate–electrolyte beverages with water [83]. This enhanced recovery was likely due to the higher sodium content in the meal. Meals also provide a variety of macronutrients and micronutrients that support recovery after dehydration.

### 4.2. Clinical Rehydration Strategies

Clinical rehydration strategies are essential for managing severe dehydration and medical conditions that require specialized hydration interventions. Unlike daily rehydration strategies, which focus on routine fluid intake through beverages, such as water or sports drinks, clinical strategies are designed to address specific medical needs, aiming for the rapid restoration of fluid and electrolyte balance. The primary methods include oral rehydration therapy (ORT), intravenous therapy (IVT), and SC fluid therapy.

#### 4.2.1. Oral Rehydration Therapy (ORT)

ORT is a simple yet highly effective clinical rehydration method. When dehydration reaches a pathological level, relying on pure water can dilute electrolyte concentrations, potentially leading to a sodium imbalance known as hyponatremia. In such cases, ORT becomes essential. ORT involves the oral administration of a specially prepared solution containing water, salts, and sugars, following the WHO guidelines for oral rehydration solutions (ORSs). This therapy is commonly used to treat mild-to-moderate dehydration resulting from diarrhea or vomiting. However, ORT is not suitable for patients with more than 10% fluid loss, those unable to consume orally due to respiratory issues or unconsciousness, individuals in shock, those unable to retain fluids due to vomiting, or patients with ileus [84]. ORS is typically administered to patients with mild-to-moderate dehydration at a dosage of 50 to 100 mL/kg every 4 h during the initial rehydration phase. After this phase, it is crucial to reintroduce nutrition and fluids promptly and continue ORT to manage ongoing gastrointestinal losses throughout the maintenance phase [84].

ORS is globally recognized as an essential oral supplement, which is particularly effective in treating diarrhea—a leading cause of dehydration and mortality worldwide. Endorsed by the WHO, ORS is a vital tool against the severe impacts of diarrheal diseases, especially in vulnerable populations, such as infants and young children. The solution is carefully formulated with carbohydrates, sodium (Na^+^), potassium (K^+^), and a base like citrate or bicarbonate to restore the fluid and electrolyte balance disrupted by diarrheal episodes [85]. When ORS enters the small intestine, it remains isotonic to body fluids. This increases extracellular fluid volume without altering serum osmolality, thereby reducing the risk of brain edema. The glucose in ORS also plays a crucial role in enhancing rehydration by promoting the release of free water through metabolism [86]. Glucose also facilitates the absorption of sodium and fluids in the small intestine via the intestinal glucose–Na transporter. A previous study showed that glucose-induced sodium absorption was not affected by cyclic adenosine monophosphate-induced active Cl secretion mediated by cholera toxin, supporting the effectiveness of ORS in treating diarrhea [87]. In human subjects dehydrated by exercise, consuming ORS was shown to result in greater fluid retention compared to plain water or sports drinks, as evidenced by lower cumulative urine output and higher serum sodium concentrations during the recovery period [88].

##### Addition of Gum Arabic or Modified Tapioca Starch in ORS

Research has shown that adding gum Arabic (GA) to a basic rehydration solution (comprising glucose, K^+^, Na^+^, Cl^−^, etc.) could effectively alleviate dehydration symptoms and improve weight recovery rates in rats with diarrhea-induced dehydration [89]. GA may enhance the absorption of water and electrolytes in the intestine by suppressing nitric oxide (NO) levels. NO is involved in magnesium-induced diarrhea and cholera toxin-mediated secretion. Reducing NO local concentrations may decrease cyclic nucleotide-mediated secretion, while increasing calcium levels. Additionally, GA might promote absorption by stimulating the release of neuropeptides, such as peptide YY (PYY) and neuropeptide YY, which act as proabsorptive or antisecretory agents, further enhancing absorption through various transport routes, such as the Na-dependent glucose carrier, or by altering gut microbiota to produce beneficial changes in volatile fatty acids over time [89].

Similarly, studies have demonstrated that adding 5.0 g/L of the modified tapioca starch Textra™ (TX) to ORS improved the absorption of water, glucose, sodium, and potassium ions in rats with diarrhea-induced dehydration, leading to significantly faster weight recovery [90]. Although the precise mechanisms remain unclear, scientists propose that TX may enhance rehydration in the following applications: 1. Acting as a secretagogue, promoting the release of PYY, which, in turn, aids in water and electrolyte absorption in the intestine. This may occur through mechanisms similar to those observed with fibers and gums, such as pectin or GA [90]. 2. Enhancing the activation of the glucose transporter 1 (GLUT1), thereby increasing the absorption of glucose and sodium. This process could involve recruiting GLUT1 transporters to the apical plasma membrane or stimulating GLUT1 mRNA synthesis [90].

These findings suggest that incorporating specific polysaccharides into ORSs can significantly enhance their effectiveness in treating dehydration. However, these applications are currently limited to animal studies, and further long-term research is needed to assess their safety and efficacy in humans.

#### 4.2.2. Intravenous Rehydration Therapy (IVT)

IVT is a critical method for directly administering fluids and medications into the bloodstream, providing a rapid and efficient way to rehydrate patients [91]. IVT is often the preferred treatment for severe dehydration, hypovolemic shock, or situations where ORT is not feasible. IV fluids are generally classified into two main types: crystalloid solutions and colloid solutions. While colloid solutions are commonly used as plasma substitutes to manage hypovolemia, no evidence exists to support their use in rehydration for dehydration recovery. Therefore, this section will focus exclusively on crystalloid solutions for rehydration.

Crystalloid solutions are widely used as IV fluids, consisting primarily of water mixed with small solutes, such as electrolytes and glucose [92]. These solutions are the preferred choice for fluid resuscitation in cases of hypovolemia, hemorrhage, sepsis, and dehydration [93]. Crystalloids are categorized based on their osmolality into three types: hypotonic, isotonic, and hypertonic solutions. Hypertonic crystalloids are generally not recommended for treating dehydration due to their negative effects on cell hydration. In contrast, hypotonic crystalloids reduce the osmolality of extracellular fluid and promote water movement into the intracellular compartment. These solutions are often used as maintenance fluids to meet daily needs for water, glucose, and electrolytes or to treat hypovolemia in patients with hypernatremia [94].

Isotonic crystalloid solutions are particularly effective for treating dehydration. Their distribution between the vascular and interstitial spaces closely mirrors the relative sizes of these compartments, with approximately 25% remaining in the vascular space and 75% dispersing into the interstitial space. This property allows isotonic crystalloids to expand the intravascular volume without significantly altering ion concentrations or causing major fluid shifts between the intracellular, intravascular, and interstitial spaces. Consequently, isotonic crystalloids are widely used to treat conditions, such as dehydration, bleeding, vomiting, diarrhea, and exudative diseases [95]. The most frequently used isotonic crystalloid solutions for rehydration are 0.9% NaCl solution (normal saline) and Ringer’s lactate solution.

Normal saline, 0.9% NaCl solution, is frequently used in clinical settings to replenish fluid deficits due to conditions, such as gastroenteritis and diarrhea [96]. It is also used to counter dehydration caused by water loss, fever, and hypernatremia induced by hyperventilation [97]. The isotonic nature of normal saline makes it suitable for the parenteral replacement of chloride losses that match or exceed sodium losses. Each 100 mL of 0.9% NaCl injection USP contains 15.4 mEq of Na^+^ ions and 15.4 mEq of Cl^-^ ions, with an osmolarity of 308 mOsmol/L and a pH range of 4.5 to 7 [98]. However, administering more than 1 L of isotonic (0.9%) NaCl per day can result in excessive sodium and chloride intake, potentially leading to hypernatremia and hyperchloremic metabolic acidosis. Therefore, the electrolyte levels in patients receiving large volumes of normal saline should be closely monitored [99].

Ringer’s lactate solution, also known as lactated Ringer’s solution, is an isotonic crystalloid fluid commonly used for rehydration in clinical settings. Ringer’s is administered intravenously and contains sodium, chloride, potassium, calcium, and lactate in the form of sodium lactate, with an osmolarity of approximately 273 mOsm/L and a pH of around 6.5 [95]. In clinical practice, the choice to administer Ringer’s solution should be tailored to the patient’s specific medical condition, as it works by expanding the vascular volume and increasing preload, thereby improving perfusion and aiding volume recovery after dehydration [100]. Additionally, Ringer’s lactate acts as a buffering solution, helping to maintain the body’s pH balance.

#### 4.2.3. Subcutaneous Fluid Therapy or Hypodermoclysis (HDC)

SC fluid therapy, or HDC, involves the absorption of fluids from the SC tissue into the circulatory system through diffusion and perfusion [101]. It is primarily used to treat dehydration in elderly patients, those with chronic illnesses, and individuals who have difficulty with oral or IV fluid administration. This approach is well-suited for long-term hydration needs, managing mild to moderate dehydration, and providing comfort to patients in palliative care who cannot tolerate other hydration methods [102,103]. HDC involves the slow infusion of fluids over several hours, facilitating a gradual and safe rehydration process.

**Figure 3:**
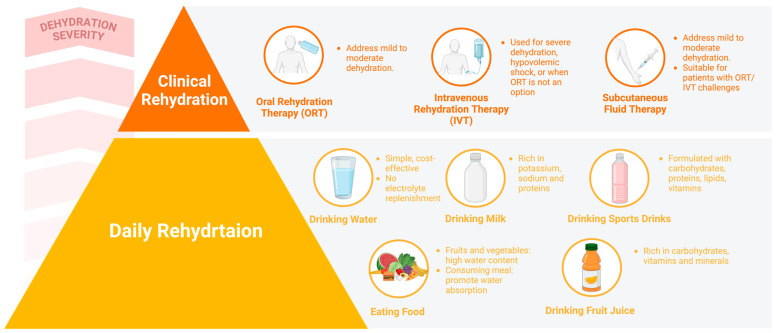
Application of rehydration strategies based on different dehydration severity.

Compared to IV hydration, HDC offers several benefits, including reduced discomfort, lower costs, fewer side effects, and a decreased likelihood of potentially serious complications. However, SC hydration does have limitations, such as the restricted volume of administered fluid, a slower delivery rate, and potential issues, such as local discomfort, swelling, and incomplete absorption [104]. Healthcare professionals should carefully consider the patient’s medical history, clinical condition, and specific therapeutic needs when deciding whether to use SC hydration (Figure 3).

### 4.3. Strategies Targeting Populations

Rehydration strategies are not universally applicable across all demographics, as different populations have varying physiological needs and vulnerabilities. In particular, athletes, elderly individuals, and pediatric patients face distinct challenges when it comes to fluid balance and recovery from dehydration. This section provides a detailed discussion of the specific benefits and potential risks associated with various rehydration methods tailored to these groups.

#### 4.3.1. Athletes

Excessive sweating during exercise, particularly in hot conditions, significantly disrupts fluid balance. This fluid loss increases physiological and perceptual strain, impairs endurance performance, and raises the risk of exertional heat illness, highlighting the need for effective hydration management in athletes [105].

Athletes typically rehydrate through the oral consumption of individualized sports drinks, which is divided into three key phases: pre-exercise, during exercise, and postexercise. Pre-exercise hydration aims to correct any existing fluid and electrolyte deficits, ensuring normal urine output before activity begins [105,106]. It is recommended that athletes consume fluids slowly at least 4 h before exercise (5–7 mL/kg). Consuming sodium-rich drinks (20–50 mEq/L) or small amounts of salty foods can stimulate thirst, improve fluid retention, and reduce the risk of overhydration or hyponatremia during exercise [106].

During exercise, maintaining hydration helps prevent excessive dehydration and associated declines in performance. Fluid intake should be adjusted based on individual sweat rates, exercise duration, intensity, and environmental conditions [106]. Athletes are encouraged to monitor body weight changes over exercise to determine appropriate fluid replacement [105,107]. The composition of rehydration fluids should also be tailored to individual needs, typically containing 20–30 mEq/L of sodium, 2–5 mEq/L of potassium, and 5–10% carbohydrates [106]. The fluid consumption rate should generally remain below 700 mL/h during endurance events to minimize the risk of hyponatremia [107].

After exercise, hydration can be restored with regular food and drinks, which provide water, electrolytes, and carbohydrates [106]. For those seeking rapid recovery from dehydration, approximately 1.5 L of fluid per kg of BWL should be consumed gradually, rather than all at once, to promote effective rehydration [106].

Athletes with a BWL > 4% should seek medical advice for personalized clinical rehydration strategies [107]. In cases of severe dehydration (BWL > 7%), where symptoms, such as nausea, vomiting, or diarrhea, are present IVT may be necessary. However, research suggests that, in most cases, IVT offers no advantage over oral rehydration for replenishing fluids and electrolytes [108]. Therefore, current research does not support the use of IVT for rehydration when athletes are capable of tolerating oral fluids [109].

#### 4.3.2. Elderly Patients

Dehydration is a common chronic health condition among apparently healthy older adults, particularly those in long-term care or who are hospitalized, often due to inadequate fluid intake [110]. As people age, the risk of dehydration increases, making the elderly a particularly vulnerable group [7,8]. Dehydration in older adults can lead to serious health consequences. Studies have shown that elevated serum osmolality in older adults is associated with an increased risk of mortality [111]. Additionally, dehydration can impair cognitive function and increase the risk of metabolic and kidney diseases [112]. Therefore, developing rehydration strategies specifically tailored to the elderly is crucial.

To prevent dehydration in older adults, the 2022 ESPEN guidelines recommend that elderly individuals consume adequate fluids or hydrating beverages based on personal preference, such as tea, coffee, fruit juices, or low-alcohol beverages containing less than 4% alcohol [113]. Additionally, eating fruits and vegetables, which are high in water, vitamins, and salts, can help prevent dehydration [114].

When treating dehydration in older adults, oral rehydration is the preferred approach if symptoms are not severe and gradual rehydration is possible. In cases of severe dehydration requiring urgent medical intervention, IVT is necessary to quickly restore fluid balance [114]. However, IVT should be carefully balanced against potential risks and complications and prescribed by a healthcare professional [114]. Another effective rehydration method for the elderly is HDC, where fluids are administered via one or two SC infusions. This low-cost treatment, with minimal side effects, can increase fluid intake by approximately 3 L per day [115]. Due to its simplicity, HDC is a practical option not only for elderly individuals in nursing homes but also for those living at home, especially in settings where IVT expertise is limited [114].

#### 4.3.3. Pediatric Patients

Dehydration is a leading global cause of pediatric morbidity and mortality, with diarrheal diseases and dehydration responsible for 14–30% of deaths among infants and young children worldwide [116]. Infants and young children are particularly vulnerable due to their higher metabolic rates, inability to express their needs or self-hydrate, and unnoticed fluid losses [117]. As a result, effective rehydration strategies for pediatric patients are critical.

For children with mild to moderate dehydration, the WHO recommends ORT as the preferred treatment method to restore normal blood volume [118]. ORT has been shown to be as effective as IVT for pediatric patients, with no significant differences in treatment failure or hospitalization rates [119]. Moreover, ORT is more affordable, requires less technical expertise, reduces the risk of complications, and allows nonmedical caregivers to participate in the treatment process [120]. Commercially prepared ORT solutions are preferred over homemade versions to minimize preparation errors [121]. To prevent vomiting, administering 5 mL of fluid orally every 1–2 min is recommended; although, this approach can be labor-intensive. Alternatively, giving a 50–100 mL/kg volume over 3–4 h is also effective for treating mild to moderate dehydration. For children under 2 years old, an additional 50–100 mL of fluid should be provided after each episode of vomiting or diarrhea. In cases where children refuse fluids or are unable to drink, nasogastric tubes may be used. As vomiting subsides, small amounts of solid food can be reintroduced, gradually returning to a normal diet according to the child’s age. Continuous monitoring of hydration status and replacing ongoing fluid losses hourly are essential during this phase [120].

For severe dehydration, IVT is required to stabilize blood volume. The recommended treatment involves administering 20 mL/kg of isotonic crystalloid solution, such as normal saline or lactated Ringer’s solution, over 10–15 min [122]. Other fluids are not recommended for pediatric volume resuscitation. Continuous monitoring of the patient’s pulse strength, electrolyte levels, capillary refill time, mental status, and urine output is critical throughout the treatment. After resuscitation and the restoration of normal electrolyte levels, the child should receive 100 mL/kg of ORT solution over 4 h [123].

### 4.4. The Mechanisms by Which Certain Nutrients Enhance Rehydration

The previously discussed rehydration strategies clearly indicate that certain nutrients significantly enhance rehydration. In this section, we will examine the mechanisms by which four key nutrients—Na^+^ ions, K^+^ ions, carbohydrates, and proteins—contribute to effective rehydration.

#### 4.4.1. Sodium and Potassium Ions

Adding electrolytes, particularly sodium, to beverages enhances fluid retention, leading to rapid rehydration, especially after significant electrolyte loss from activities, such as intense exercise [83,124]. The fundamental function of electrolytes in water retention is to influence the distribution of water between the intracellular and extracellular compartments by maintaining the osmotic pressure of the extracellular fluid [125]. Sodium, the primary ion in extracellular fluid, plays a vital role in facilitating quick rehydration. When sodium and water are consumed together after dehydration, some sodium remains in the vascular space, preventing the drop in plasma osmolality that occurs with plain water intake. This helps maintain stable levels of vasopressin and aldosterone, as well as consistent diuresis, avoiding a negative fluid balance. Vasopressin, secreted by the posterior pituitary gland in response to increased blood osmolarity, reduces urine production by increasing water reabsorption in the kidneys. Sodium reabsorption in the renal tubules, which is often coupled with water reabsorption, is further facilitated by the sodium–potassium pump. This pump moves sodium into the bloodstream, creating an osmotic gradient that drives water reabsorption, a process regulated by aldosterone, which responds to low blood volume or sodium levels [126,127,128]. Additionally, maintaining plasma osmolality and sodium concentration can trigger thirst, promoting adequate fluid intake [124]. Sodium also enhances water absorption in the small intestine [71]. For optimal fluid balance, the sodium concentration in rehydration beverages should match or exceed the amount of sodium lost through sweat [129].

In contrast, potassium, the primary ion in intracellular fluid, also supports rehydration, particularly within cells. A study of dehydrated rats observed that beverages containing potassium chloride (KCl) were often more effective at restoring intracellular fluid volume than those containing NaCl [130]. Potassium in beverages can aid in maintaining intracellular fluid retention, supporting rapid rehydration [124].

#### 4.4.2. Carbohydrates and Proteins

Research has shown that rehydration drinks with higher carbohydrate content (6% to 10%) are associated with greater fluid retention during recovery after exercise [131,132]. The most widely accepted explanation for how glucose enhances water absorption is the coupled transport hypothesis. In conditions where intestinal sodium absorption is compromised, such as diarrhea, administering an oral saline solution may not be effective. Without sodium absorption, water cannot be absorbed, and excess sodium in the intestinal lumen may exacerbate dehydration by increasing water secretion. However, adding glucose (dextrose) to the solution initiates a different mechanism. Glucose molecules are absorbed through the intestinal wall, carrying sodium with them via a cotransport mechanism that operates on a 1:1 ratio, wherein one glucose molecule cotransports one sodium ion. While glucose does not directly transport water, the increased sodium concentration across the intestinal wall pulls water through by osmosis [133,134]. Additionally, glucose can stimulate active sodium transport in the human jejunum [135].

**Figure 4:**
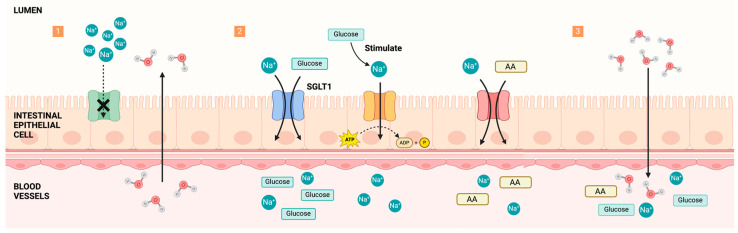
Water absorption enhancement mechanisms of glucose and amino acids.

Numerous studies have investigated the role of protein-containing beverages in rehydration. The findings indicate that beverages combining carbohydrates with milk protein enhance rehydration more effectively than carbohydrate-only beverages of equivalent energy content [31,77]. Adding 1.5% whey protein to carbohydrate–electrolyte beverages further enhances rehydration [136]. Beyond their impact on fluid balance, protein-containing beverages consumed postexercise can promote muscle protein synthesis, accelerating recovery [137]. Specific amino acids have also been shown to increase water and sodium absorption in the intestine [138,139]. Besides glucose, amino acids, dipeptides, and tripeptides can also cotransport sodium. Amino acids and sodium are coupled in a 1:1 molar ratio, while dipeptides and sodium are coupled in approximately a 1:2 molar ratio. Rehydration solutions containing both glucose and amino acids demonstrate a superior rehydration effect, indicating an additive benefit [140] (Figure 4).

Unlike amino acids and glucose, which are cotransported with sodium to promote water absorption in the small intestine, fat does not have a direct mechanism for promoting rehydration. Fat is absorbed primarily through the lymphatic system via emulsification, enzymatic digestion, and chylomicrons, rather than entering the bloodstream directly like other nutrients involved in rehydration. Although polyunsaturated fatty acids have been included in rehydration fluids to reduce oxidative stress and support recovery, there is no direct evidence that fats improve rehydration.

## 5. Conclusions

In vivo dehydration models are primarily established through four methods: fluid restriction, exercise, thermal exposure, and chemical induction. These approaches allow researchers to replicate dehydration under controlled settings, enhancing our understanding of its physiological and pathological effects. Rehydration in these models can be administered through various routes, including oral, intestinal, IV, SC, or IP methods, each offering a way to assess the effectiveness of different rehydration strategies. Simple interventions, such as drinking plain water, consuming beverages, and eating food, are typically sufficient to restore fluid balance and address mild-to-moderate daily dehydration. In contrast, pathological dehydration necessitates clinical interventions, including ORT, IVT, and SC fluid therapy, to safeguard patient health. For vulnerable groups, such as athletes, the elderly, and pediatric patients, rehydration must be personalized to match their unique physiological conditions. These insights pave the way for developing personalized rehydration strategies tailored to specific situations, particularly in environments where dehydration is a critical concern, such as in elderly care or high-performance sports.

Despite advancements, the absence of standardized dehydration protocols across studies hinders the ability to compare results or determine optimal rehydration strategies. Additionally, no connection between specific dehydration models and their corresponding rehydration administration and protocols has been found, which complicates the rehydration process for these models. The cellular and molecular mechanisms underlying dehydration and rehydration are also poorly understood, limiting the development of targeted therapies. Addressing these gaps will require more precise experimental methods and the integration of emerging technologies. Wearable hydration sensors, for instance, could offer continuous, real-time fluid balance monitoring, improving dehydration management in both everyday and clinical settings. Additionally, the discovery and validation of new dehydration biomarkers could enhance diagnosis and treatment, particularly for vulnerable populations.

## 6. Future Perspectives

Future research should prioritize understanding the molecular and cellular responses to dehydration and rehydration, including signaling pathways, osmotic stress responses, and tissue-specific adaptations. High-resolution imaging techniques, such as advanced MRI or PET scans, could provide deeper insights into dehydration’s structural and functional impacts on organs. Furthermore, developing more accurate animal and cellular models that better replicate human dehydration could help bridge the gap between basic research and clinical practice.

## Figures and Tables

**Table 1:** Evaluation of mouse dehydration by appearance and attitude [16].

Appearance Score		Attitude Score	
5	Normal; skin tent and posture appear normal	5	Normal; exhibits activity in the cage prior to and during handling
4	Noticeable skin tent on the dorsum	4	Reduced activity, yet alert and responsive to handling
3	Hunched posture with piloerection and moderate skin tent	3	Lethargic, diminished resistance to handling
2	Eyes appear sunken, with severe piloerection and pronounced skin tent	2	Unresponsive; only moves when physically touched
1	Unable to right itself	1	Fails to flee when a hand is presented in the cage

**Table 2:** Dehydration assessment of dogs [20].

Dehydration Level	Physical Exam Findings
Euhydrated	Normal hydration status
Mild (~5%)	Minimal loss of skin turgor, semidry mucous membranes, normal eye appearance
Moderate (~8%)	Moderate loss of skin turgor, dry mucous membranes, sunken eyes (enophthalmos), weak and rapid pulses
Severe (>10%)	Considerable loss of skin turgor, extremely dry mucous membranes, severe enophthalmos, tachycardia, weak/thready pulses, altered consciousness, hypotension

**Table 3:** Summary of induction methods of different dehydration models.

Method of Dehydration Induction	Method/Instruments	Subject	Description	Advantages	Disadvantages	Indicators	References
Fluid-restriction-induced dehydration	Acute dehydration	Human volunteers	Continuous water restriction Human: 13 h (1.0% BWL), 24 h (1.8% BWL), 37 h (2.7% BWL)	Straightforward Affordable More reference value	Time-consuming Ethical considerations	Thirst, headache, body weight, plasma volume, serum sodium, serum chloride, serum osmolality, urine volume, urine sodium, urine potassium, urine chloride, urine osmolality, angiotensin II	[24]
		Animals	Continuous water restriction Rats: 18–24 h (6% BWL), 36–48 h (6–10% BWL), 66–72 h (>10% BWL) Mice: 24 h (12% BWL), 48 h (18% BWL) Dogs: 12–24 h	Straightforward Affordable	Time-consuming Difficult to control precisely	Food intake, body weight, attitude and appearance scores, plasma volume, plasma osmolality, plasma sodium, plasma corticosterone, packed-cell volume (PCV), total plasma protein, plasma renin activity (PRA), hematocrit, blood urea nitrogen (BUN), platelet count, urinary sodium, urinary potassium, urinary osmolality, neutrophil, lymphocyte, white blood cell (WBC) count	[14,15,16,17,18,19,20]
	Chronic dehydration		1. Limiting the daily water intake Rats/Mice: 50/75% daily water intake for 7 days 2. Intermittent water restriction Rats: water deprivation for 15 h each evening over a 20-day period/10 h of water restriction every 24 h, 5 days a week, over 4 weeks				[16,21,22,23]
Exercise-induced dehydration	Treadmills	Animals	Rats: acclimated to treadmill for 3 days before, four sets of exercises at a rate of 25 m/min, 2 min interval between each set	Precise control over exercise duration and intensity	Possibility of interference among test subjects Reduced activity levels Equipment required	Sodium, potassium, chloride, calcium, phosphorus, magnesium, creatine phosphokinase (CPK), lactate dehydrogenase (LDH), serum urea, creatinine, aldosterone	[26]
	Treadmill/stationary bike/friction-braked cycle ergometer/Monark cycle ergometer/elliptical machine	Human volunteers	1. Fixed-intensity and duration exercise protocols Cycled for 1–1.5 h at 50–60% of their maximum heart rate and 30–32 °C/three sets of 25 min intermittent-intensity exercises on a treadmill, stationary bike, and elliptical machine, with 5 min rest periods between sets. 2. Exercise to a predetermined percentage of body weight loss 1.6% BWL: exercised on a friction-braked cycle ergometer at an intensity of 2 W/kg body mass in a 35 °C environment, with each session lasting 10 min, followed by a 5 min rest. 3–5% BWL: cycled on a Monark cycle ergometer at 60 rpm in a 38 °C climate chamber 3. Combine fluid restriction with exercise-induced dehydration Walked on a motorized treadmill at 5.6–6.4 km/h on a 2% incline for 25 min, followed by a 5 min rest, repeating this cycle six times. Fluid intake was restricted for 14 h before	More reference value More cooperative	Ethical considerations Equipment required	Body weight, thirst perception (TH) scale, thirst sensation scale (TSS), urine-specific gravity, urine color, saliva flow rate, urine volume, urine osmolality, serum osmolality, tear fluid osmolality, sweat sodium concentration	[27,28,29,30,31,32,33,34]
Thermal dehydration	High temperature	Animals	Exposing animals to higher room temperatures Rats: 40 °C for 0–4 h without water provided/infrared lamp to raise the colonic temperature by 0.05 °C/min for 60 min or until it reached 41.5 °C Mice: 37 °C and 15–20% relative humidity without water provided	Straightforward	Cause undue harm	Body weight, water intake, plasma osmolality, plasma sodium, plasma potassium, hematocrit, plasma protein, urine output	[35,36,37,38]
Chemical-induced dehydration	Saline treatment	Animals	1. Subcutaneous injection Mice: 3% or 6% weight/volume, 0.5 mL/mouse 2. Intracerebroventricular injection Mice: 600 mM/μL, 2 μL/mouse 3. Intraperitoneal injection Mice: 10 mL of 1 M NaCl/kg b.w. 4. Oral treatment Gavage (rats): 2 mL of 2 mol/L NaCl/3 mL of 1.5% NaCl three times daily for 20 days Consume water containing 2% NaCl for 6–8 days (rats)	Less time-consuming (except for long-term oral treatment) Less confound variables	Difficult to operate Cause undue harm	Water intake, plasma sodium, plasma osmolality	[39,40,41,42,43,44,45]
	Isoproterenol treatment		Subcutaneous injection Mice: 15–400 μg/kg			Water intake	[39,40]
	Polyethylene glycol treatment		Subcutaneous injection Mice: 25% wt./vol, 1 mL/mouse			Water intake	[39,40]
	Angiotensin ll treatment		Intracerebroventricular injection Mice: 3.1–100 ng/2 μL, 2 μL/mouse			Water intake	[39,40,46,47,48,49,50]
	Furosemide treatment		1. Intraperitoneal injection Rabbits: 50 mg/mL, 5 mg/kg 2. Intravenous injection Rabbits: 5 mg/kg Rats: intravenous injection of 8 mg/kg furosemide following 4.2% hypertonic saline (3.2 mL/100 g)			Body weight, urine output, plasma volume, plasma sodium, plasma potassium, plasma chloride, hemoglobin, packed cell volume, total plasma protein, creatinine, blood urea nitrogen, brain water content, pulmonary water content, muscle water content, small bowel water content	[52,53,54]
	Captopril treatment		Chronic addition of captopril to the drinking water Mice: 0.5–1.0 mg/mL	Easy to operate	Time-consuming, imprecise control of intake	Water intake	[39]
